# Change in Nutrition Behavior After Participating in an Obesity-Related Cancer Education Program in El Paso, Texas.

**DOI:** 10.1177/10732748241261567

**Published:** 2024-06-07

**Authors:** Mary Miller, Roy Valenzuela, Jennifer J. Salinas

**Affiliations:** 1 L. Frederick Francis Graduate School of Biomedical Sciences, Texas Tech University Health Sciences Center, El Paso, TX, USA; 2 Department of Social Work, College of Health Sciences, 12337 The University of Texas at EL Paso, El Paso, TX, USA

**Keywords:** nutrition, cancer, education, outreach, evidence-based, Mexican Americans

## Abstract

**Background:**

Roughly 25% of the U.S- Border city, El Paso, Texas is obese. Obesity is a major risk factor for 13 cancers. Cancer is the leading cause of death in El Paso. Therefore, there is a growing urgency to implement evidence-based programs that support behavioral change that helps curb the impact of obesity in El Paso and the U.S.-Mexico border region.

**Purpose:**

This study aimed to assess the effectiveness of an obesity-related cancer prevention program (Pasos Para Prevenir Cancer (PPPC) on changes in participant nutrition behaviors.

**Methods:**

Culturally tailored, theory-based education was provided to adults through the PPPC program. A total of 256 PPPC participants agreed to take part in our program evaluation. Participants were asked to complete a survey at baseline and 6 months after they completed the program. Session included topics on obesity-related cancers, assessing your obesity risk, measuring body fat, SMART goal setting, and how to find the right type of physical activity. For this report we used the Food Frequency Questionnaire (FFQ) data to assess changes between baseline and six months. We also used perceived dietary barriers as moderators on the relationship between program participation and nutrition behaviors.

**Results:**

Most participants (92.2%) identified as being of Mexican American descent, were between the ages of 41-75 years of age (n = 165) and identified as females (n = 225). 48.1% of the participants were born in Mexico while 50.4% were born in the U.S. Approximately 35-51% of participants improved and sustained their intake of healthier foods at 6 month follow up. Specifically, there was a statistically significant shift from higher fat and sugar content foods to light and low-fat foods, and fruits and vegetables. Participants also increased their consumption of ground chicken, lean red meat, and seafood. A key modifier in this relationship is perceived health risk.

**Conclusion:**

Latinos on the U.S.-Mexico border ascribe to a healthy living mindset. In general, they frequently eat fruits and vegetables. Participation in PPPC increased perceived barriers to healthy living around cost and convenience and enhanced decision-making around healthier options. Participants responded to our adapted evidence-based program resulting in sustained changes in nutrition behaviors. Using adapted evidence-based strategies developed outside of the U.S.-Mexico border region is a feasible approach to address persist health disparities.

## Introduction

Socioeconomic challenges create barriers to healthy eating and active living, thereby leading to health disparities in obesity, chronic diseases and cancers.^1,2^ As a result, there is a high concentration of MA in labor-intensive occupations such as agriculture, construction, and hospitality.^3,4^ Jobs in these sectors have significant pay disadvantages compared to the jobs commonly affiliated with advanced education.^2-4^ MA earn on average 60% less than NHW.^2-6^ Being low-income creates barriers for MA to maintain healthy nutrition and physical activity behaviors, oftentimes forcing them to choose between a healthy lifestyle and safe affordable housing or medications that prevent the onset of more debilitating disease and/or cancer.^6-10^

In addition, MA face barriers to health care access. Historically, systemic inequities in health care have created disproportional clinical outcomes for MA compared to Non-Hispanic Whites (NHW).^11-15^ Provider bias, cultural differences, and structural inequalities such as inaccessibility to interpretations services affect 30% of MA, increasing their chance of experiencing an adverse healthcare-related event that results in injury by 40% compared to NHW.^12,16^ These medical experiences have led many MA to lose trust in health care professionals leading to lower medication adherence and lower preventive care uptake compared to NHW.^5,11-16^ Socioeconomic disparities coupled with barriers to health care services are fundamental social determinants of health that drive health disparities observed among MAs particularly those residing in the U.S.-Mexico border region. Therefore, evidence-based strategies that are community-based are key to addressing persistent health disparities. In this study, we sought to evaluate an adapted evidence-based program, Pasos Para Prevenir Cancer (PPPC), to determine to the effectiveness of strategies developed outside the U.S.-Mexico border region in changing nutrition behaviors among Mas residing in El Paso, Texas.^7,8,17-27^

Obesity is a significant risk factor for 13 types of cancers.^28-30^ Approximately 25% of adults residing in the U.S.-Mexico border city of El Paso are obese.^31^ Evidence-based obesity prevention programs have been shown to reduce the impact of obesity-related diseases.^18,32^ However, prevention strategies from these programs have not been widely adopted on U.S.-Mexico border where Mexican American (MA) are the majority.^7,19,20^

Pasos Para Prevenir Cancer (PPPC), is an adapted evidence-based obesity-related cancer prevention program implemented in El Paso, Texas.^33^ The program’s topics included education on 13 obesity related cancers, nutrition, physical activity, and culturally tailored materials for the MA population.^33^ Program participants attended up to five sessions of classes.^33^ The purpose of this study is to determine if participation in PPPC is associated with sustained nutritional behavioral change, and if perceived nutritional barriers modifieds this relationship.

## Methodology

### Participants

Participants for this evaluation study were recruited from PPPC education sessions. Participants were invited to participate in the evaluation study upon completion of the program. Data was collected at baseline, 6-months and 12-months in this study. We report in this study results from baseline and 6-months. If program participants agreed to participate, they were sent a survey link from REDCap. Informed consent was completed online and participants would only receive the survey if they agreed to participate by responding ‘yes’ or ‘no’ on the REDCap-based consent form. Participants were included in the evaluation study if they were at least 18 years old and not pregnant. A total of 256 participants were surveyed at baseline and six months. Missing data was managed using the intent to treat.

## Measurement

### Evaluation Outcome

The Food Frequency Questionnaire (FFQ) was used to collect nutrition data at baseline and 6-months. The FFQ, is a 30-to-60-minute, 125 food and beverage line items and a 26 dietary supplement questionnaire. This questionnaire, which was validated in English and Spanish, asked participants to rank nutritional behaviors on a Likert scale to measure the frequency of consumption of various foods and portion sizes.^34^ Frequency of consumption ranged from 0 and 10. “Never” was represented by 0 and 10 represented “2 or more times per day.” From the FFQ data we created a categorical variable to reflect change between baseline and 6 months in food type consumption. The outcomes of interest included were fruits, vegetables, healthier dairy, healthier meat, and healthier fats. Baseline values were subtracted from 6 month values to create never or decreased in consumption a food type, moderate increase in consumption, and significant increase.^6-10^

### Independent Variables

Our primary independent variables were baseline perceptions of nutrition behavior barriers. Participants were asked to assess their perception of barriers to eating healthy on a Likert scale. Questions asked that were included in our analysis were: “I think that the long-term risks of unhealthy nutrition are heart disease, diabetes, cancer, and poor bone health,” “I think it is difficult to have my family consume a healthy, balanced diet and to engage in frequent physical activities, ” and “I think healthy foods taste good.”

We created dichotomous variables in each perception behavior barrier variable at baseline where 1 = agree/strongly agree and 0 = disagree/strongly disagree. by placing participants who chose to scale between 0 and 1 into Group 1 and participants who chose to scale between 2 and 4 in Group 0.

### Covariates

Demographic covariates included age (18 years and over), gender, ethnicity (Mexican American/not Mexican American), marital status (married or single), education level (high school diploma or above), birthplace (U.S. born or foreign-born), and employment status (employed for wages or unemployed/retired/unable to work).

### Data Analysis

We conducted univariate analysis to observe trends in the data and to address outliers and missing data. To assess research question 1, we used distribution analysis to assess changes in nutritional behavior between baseline and 6 months. To address our second research question, OLS regression analysis was used to predict the change in food type variety by baseline perceived barriers variables.

## Results

Table 1 presents the demographic characteristics of our sample (see Table 1). There were 256 total participants, of which 92.2% were Mexican American (*n* = 236) and 7.8% were not Mexican American (*n* = 20). A total of 30.9% were between the ages of 18 and 40 (*n* = 79); 64.5% were aged 41-75 (*n* = 165); and 4.7% were aged 76-92 (*n* = 12), 87.9% were female (*n* = 225), 49.6% of participants were foreign-born (*n* = 127). There was 61.6% of participants who were married (n = 157) and 55.5% of participants were employed (*n* = 142). Of those who reported their income, 42.8% of participants earned under $35,000 annually (*n* = 109). However, a significant percentage did not report their income at all 21.6%. Finally, more than two-thirds of participants (62.5%) obtained a college degree or beyond (*n* = 160).

**Table 1.** Participant Demographic Characteristics (n = 256).

Variables	n	%
Ethnicity		
Mexican american	236	92.2
NOT mexican american	20	7.8
Age		
18-40	79	30.9
41-75	165	64.4
>75	12	4.7
Gender		
Male	31	12.1
Female	225	87.9
Place of birth		
Mexico Born	123	48.1
U.S born	129	50.4
Other	4	1.6
Marital status		
Married	157	61.6
Single/Other	99	38.4
Employment status		
Employed	142	55.5
Unemployed, other	114	44.5
Annual total household income		
Under $35,000	109	42.8
Over $35,000	91	35.7
Other/Not reported	56	21.6
Education		
H.S diploma or below	96	37.5
College or beyond	160	62.5

**Figure 1.**
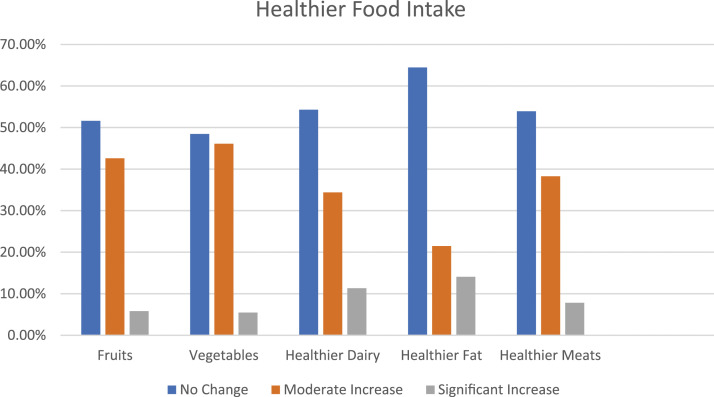
Change in consumption of healthier food intake by PPPC participants at 6 months.

Figure 1 Displays change patterns of healthier food options by food type. About 49% of participants increased their consumption of healthier foods. Approximate 40% of participants made moderate increases in their intake of healthier foods, and about 8% of participants made significant increase in their consumption of healthier foods at 6 month follow-up. Increased consumption of healthier foods was most notable in fruits, vegetables, and healthier dairy. Specifically, 42.6% of participants moderately increased their fruit consumption variety, and, about 5% of participants substantially increased their fruit consumption variety. Similarly, 46.1% of participants increased the variety and consumption of vegetables moderately and 5.5% of participants made the effort to significantly increase their vegetable consumption with the addition of 11-15 types of vegetables. Finally, 34.4% of participants increased their healthier dairy consumption by moderately adding 1-3 healthier dairy options and 11.3% increased their healthier dairy variety in the amount of 4-6 new healthier dairy types.

OLS regression modeling indicated that the perceptions of long-term health benefits were significantly associated with the increases seen in variety consumption of fruits, vegetables, and healthier dairy between baseline and six months (See Table 2). The increase in variety of fruit (β = 1.55; *P*-value = .01) and vegetable servings (β = 3.55; *P*-value = .001) was significantly associated with the perception of the long-term health benefits (See Table 2). Healthier dairy increase in intake was also significant (See Table 1) (β = 1.28; *P*-value = .004). There were no significant relationships with taste or family difficulties in observed nutrition behavior changes.

**Table 2.** OLS Regression Results Assessing Relationship Between Perception of Long-Term Health Benefits and Change in Healthier Food Variety Behaviors at 6-Months.

Perceived Barriers	Fruits	Vegetables	Healthier Dairy
Family difficult eaters	.03 (n.s.)	.05 (n.s)	.03 (n.s.)
Long term health benefit	1.75 (.009)	3.55 (.001)	1.28 (.004)
Healthy food taste	.01 (n.s.)	.04 (n.s.)	.01 (n.s.)

## Discussion

In this evaluation study, we investigated the changes in nutritional behaviors following participation in the Pasos Para Prevenir Cancer obesity-related cancer prevention program. Our findings demonstrate that PPPC participation was associated with significant increases in the variety of healthier food options. These changes were most notable in the increased variety in consumption of fruit, vegetables, and healthier dairy. Perceptions related to long-term health benefits at baseline was the only associated health belief. The findings suggest that if participants view nutrition as benefiting their health through cancer and other chronic disease prevention, they were more likely to increase the variety of fruits, vegetables and lower fat dairies.

PPPC participation is associated with sustained increase in the number of fruit, vegetable, and low fat dairy servings 6 -months after program completion. These findings are consistent with evidence-base programs tested in non-Hispanic white and non-border Latino populations and settings. ^35-39^ An important distinction here in this study is that the context in which the intervention was delivered had unique socioeconomic challenges that did not seem to impact the effect of participation in evidence-based programming developed with other populations in mind. These are promising findings for prevention efforts on the border, as most evidence-based programming is primarily tested in non-border populations.^35-39^ While this study’s focus was on nutrition, adapting evidence-based content related to preventable cancers and other conditions has potential to address persistent health disparities more affecting this population.

This study adds to our understanding of how U.S.-Mexico residing Mexican Americans perception about disease risk translates into sustained behavioral changes. Participants, who were predominantly Mexican American, who believed that nutrition was important in preventing disease were more likely to add more servings of fruits, vegetables, and lower fat dairies into their diet. However, family difficulty and taste were not significant for any of the food types. When working with Mexican American populations, cultural tailoring often focuses on the family as motivation for change, or, taste of favor cultural cuisines. These findings may suggest that tailoring may not need to focus as much on culture, but in the real risk of disease associated with certain behaviors in this context. This is an area of opportunity in intervention design and implementation studies targeting disease outcomes that share the same behavioral risk factors. Presenting information to behavioral intervention participants on global disease risk, could lead to better likelihood of sustained behavioral change and uptake of healthier nutrition and other behaviors that prevent diseases.

## Limitations

Despite our positive findings, there are some limitations. First, PPPC was implemented only in El Paso, Texas, which is one of many regions on the U.S.-Mexico border. Further studies should evaluate the effectiveness of adapted evidence-based programs in other areas of the U.S.-Mexico border. For example, there are large rural geographies that should be considered when conducting implementation research. A second limitation is that most of our sample was female. Therefore, inferences to men are limited. The city of El Paso has a Hispanic/Latino college graduate rate (28.1%) which is slightly higher than the national average for all Hispanics (20%). This is just one aspect of how El Paso and the U.S.-Mexico border is unique and not always representative of the U.S.- Hispanic population at large. With that said, a third limitation in this study is that our sample was predominantly college educated and did not represent the population of El Paso that was less educated. This was due in part to the fact that our study participants were recruited from a program that did not involve a randomized sample. Therefore, in future evaluation studies there should be an effort to focus on non-college educated Hispanics to assure that the evidence-based strategies are successful at influencing change in nutritional behaviors. A fourth limitation is that the program evaluation occurred during the Covid-19 pandemic and as a result this may have affected participants nutrition behaviors and responsiveness to our intervention. Finally, because the data collected was for the purpose of program evaluation, we used a pre-post design and did not include a control group. Future studies should include an experimental design to further evaluate the effectiveness of this adapted intervention. Related to this issue is that evaluation study samples are largely dependent on willingness of participants to take part in the study component of the program. We had 256 participants who agreed and followed through with the evaluation study, which in our opinion is a reasonably sized sample for a study a study of this kind. Generally, when we are looking at power, we are concerned about null findings as the statistical analysis may not yield differences due to the low sample size. However, in our study, with the sample size that we had, we did observe significant relationships that contribute to our understanding adaptive interventions to improve health behaviors in the U.S-Mexico border region.

### Future Directions

Despite these limitations, this evaluation study provides evidence that adapted evidence-based strategies developed in other populations to improve nutrition behaviors are feasible and effective. However, further investigation to determine effectiveness in other settings on the U.S.-Mexico border are needed.

## Conclusions

This evaluation study of PPPC, an obesity-related cancer prevention program, provides insight into the adaptations implemented in a Latino population residing on the U.S.-Mexico border. Behavioral education that is supplemented with long-term benefits of disease prevention may improve sustained behavioral change on the U.S.-Mexico and in other Latino populations.
